# Is heme metabolism a promising drug target in *Toxoplasma gondii*?

**DOI:** 10.1371/journal.ppat.1014280

**Published:** 2026-06-02

**Authors:** Zhicheng Dou

**Affiliations:** 1 Department of Biological Sciences, Clemson University, Clemson, South Carolina, United States of America; 2 Eukaryotic Pathogens Innovation Center, Clemson University, Clemson, South Carolina, United States of America; University of Wisconsin Medical School, UNITED STATES OF AMERICA

*Toxoplasma gondii* is one of the most prevalent parasitic infections worldwide, with seroprevalence estimates indicating that up to one-third of the global population has been exposed [1]. Although infection is typically asymptomatic in immunocompetent individuals, *Toxoplasma* poses a severe, potentially life-threatening risk to immunocompromised patients, including organ transplant recipients, individuals undergoing chemotherapy, and people living with HIV/AIDS [1]. During pregnancy, congenital transmission can cause devastating harm to the developing fetus [2]. The current frontline treatment for acute toxoplasmosis uses a combination of pyrimethamine and sulfadiazine, which carries significant side effects in some patients [3]. In addition, pyrimethamine exhibits potential teratogenicity [1]. Spiramycin, a macrolide antibiotic used during pregnancy, cannot cross the placenta and is therefore ineffective for treating the infected fetus [2]. Critically, none of these commercially available drugs eliminates chronic *Toxoplasma* infections. There is therefore an urgent need to identify novel drug targets within the parasite.

As an obligate intracellular parasite, *Toxoplasma* relies on a combination of *de novo* biosynthesis and host salvage to obtain essential nutrients [4,5]. Notably, the parasite maintains a complete *de novo* heme biosynthesis pathway, yet its spatial organization differs substantially from that of the mammalian host [6–9]. These metabolic differences provide a solid foundation for the development of parasite-specific therapeutics. This Pearls article reviews recent progress in understanding heme metabolism in *Toxoplasma* and evaluates its potential as a drug target.

## How does the parasite pathway differ from its mammalian counterpart?

**Fig 1:**
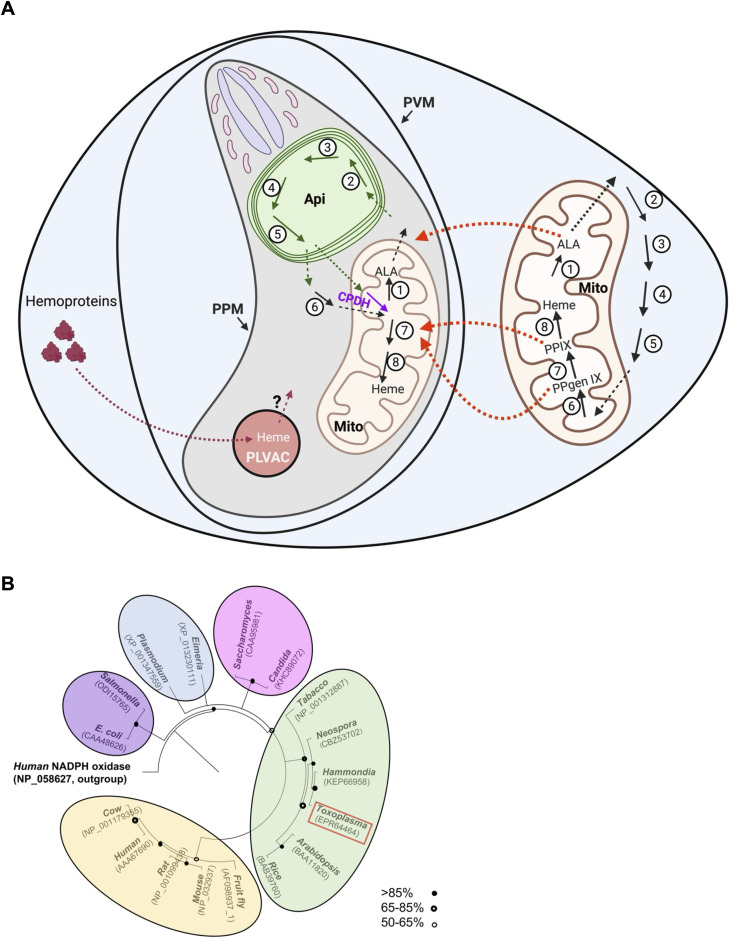
Heme acquisition in *Toxoplasma gondii.* **(A)** In *Toxoplasma*, the heme biosynthetic pathway is distributed across three compartments: the mitochondrion (Mito; ALAS, step 1; PPO, step 7; and FECH, step 8), the cytoplasm (CPOX, step 6), and the apicoplast (Api; PBGS, step 2; PBGD, step 3; UROS, step 4; and UROD, step 5). In contrast, the mammalian host pathway is shared between the mitochondrion (steps 1, 6, 7, and 8) and the cytoplasm (steps 2–5). The parasite also encodes a mitochondrion-residing, oxygen-independent enzyme, CPDH (purple arrow), which catalyzes the same reaction as CPOX. Current evidence suggests that the parasite can salvage heme intermediates from the host cell (orange dashed arrows), thereby rescuing growth defects caused by disruptions in parasite heme biosynthesis; however, intact heme itself does not appear to be salvaged from the host. Because *Toxoplasma* can ingest host proteins via its plant-like vacuolar compartment (PLVAC) for nutrient acquisition, it has been hypothesized that the parasite may also obtain heme from host-derived hemoproteins through this route (crimson dashed arrows). However, the relevant heme transporter(s) within the PLVAC remain unidentified, and this pathway is still speculative. Green and black dashed arrows indicate trafficking of heme intermediates across parasite organelles. **(B**) Phylogenetic analysis of TgPPO. Figure reproduced from Bergmann *et al., PLoS Pathogens* 2020;16:e1008499. Abbreviations: Api, apicoplast; Mito, mitochondrion; PLVAC, plant-like vacuolar compartment; PVM, parasitophorous vacuole membrane; PPM, parasite plasma membrane; ALA, 5-aminolevulinic acid; ALAS, 5-aminolevulinate synthase; PBGS, porphobilinogen synthase; PBGD, porphobilinogen deaminase; UROS, uroporphyrinogen III synthase; UROD, uroporphyrinogen decarboxylase; CPOX, coproporphyrinogen oxidase; CPDH, coproporphyrinogen dehydrogenase; PPgen IX, protoporphyrinogen IX; PPO, protoporphyrinogen oxidase; PPIX, protoporphyrin IX; FECH, ferrochelatase. This figure was created with BioRender.

Like mammalian cells, *Toxoplasma gondii* encodes all eight enzymes required for *de novo* heme biosynthesis [6–8,10]. In mammals, the pathway is split between the mitochondrion (first and last three steps) and the cytoplasm (four intermediate steps). In *Toxoplasma*, four intermediate steps are localized to the apicoplast, a plastid-like organelle unique to the phylum Apicomplexa that arose through secondary endosymbiosis of a red alga [6–10], rather than the cytoplasm, representing a plant-like organizational feature (Fig 1A). Additionally, while mammalian coproporphyrinogen oxidase (CPOX) resides in the mitochondrial intermembrane space, TgCPOX is a cytosolic enzyme [6].

A further point of divergence is the presence in *Toxoplasma* of TgCPDH, an ortholog of coproporphyrinogen III dehydrogenase, which resides in the parasite’s mitochondria (Fig 1A) [8,11]. This oxygen-independent enzyme catalyzes the same reaction as TgCPOX using a radical S-adenosyl-L-methionine (SAM) mechanism for oxidation. TgCPDH is highly homologous to bacterial ortholog HemN [11]. These findings suggest that TgCPDH may sustain coproporphyrinogen III oxidation under low-oxygen conditions, thereby maintaining heme production when molecular oxygen is limiting. These structural and organizational differences between the parasite and host pathways provide a rational basis for developing parasite-selective inhibitors.

## Is the de novo heme biosynthesis pathway essential for Toxoplasma intracellular growth?

Recent genetic studies have provided definitive evidence that this pathway is essential for parasite survival [6–8]. Multiple enzymes in the pathway, including TgALAS, TgUROD, TgCPOX, and TgFECH, have proven essential or near-essential by genetic disruption, and all heme-deficient mutants exhibit dramatically attenuated virulence *in vivo* [6–8]. Among the enzymes examined, loss of protoporphyrinogen oxidase (TgPPO) had the least impact on parasite fitness, reducing growth by ~50% relative to wildtype [6]. Nevertheless, even this strain displaying a partial heme deficit still attenuates the parasite’s acute virulence. Inoculation with 100-fold higher numbers of TgPPO-deficient parasites than the wild-type lethal dose fails to cause mouse mortality [6].

Functional analyses reveal that heme deficiency strongly disrupts mitochondrial physiology. In TgUROD knockdown parasites, heme levels fell by ~90%, causing a drastic reduction in mitochondrial oxygen consumption and ATP production [7]. Expression of c-type cytochrome proteins was also remarkably reduced, consistent with a heme requirement for cytochrome maturation [7]. Deletion of TgCPOX more severely reduces heme production and even abolishes mitochondrial respiration, with a corresponding upregulation of glycolytic activity [8]. Similarly, genetic disruption of TgALAS and TgPPO also reduces heme production, albeit to different extents [6]. Intriguingly, intracellular growth rates correlate linearly with heme abundance across these mutants [6], establishing a dose-dependent relationship between pathway activity and parasite fitness.

Our recent work demonstrated that TgCPDH can functionally complement a *Salmonella* mutant lacking both HemF (an ortholog of CPOX) and HemN, restoring bacterial growth in heme-free medium [11]. Furthermore, deletion of TgCPOX leads to upregulation of *TgCPDH* transcript levels, although protein abundance remains unchanged [11]. Despite this evidence of enzymatic activity, loss of TgCPDH did not alter acute or chronic virulence in *Toxoplasma* [8], suggesting that it plays a dispensable role in parasite heme metabolism.

The capacity of several heme biosynthesis-deficient mutants to propagate in culture, despite displaying highly reduced virulence in mice, likely reflects partial compensation by host-derived precursor scavenging that is sufficient in nutrient-rich media but inadequate to sustain infection in the host environment, indicating that *in vitro* fitness underestimates *in vivo* pathway dependence. Taken together, these findings demonstrate that *de novo* heme biosynthesis is indispensable for *Toxoplasma* mitochondrial function and intracellular survival, and thus represents an attractive target for therapeutic intervention.

## Can the parasites salvage heme or heme biosynthetic intermediates from the host?

Free heme is cytotoxic and is strictly sequestered by proteins within the cellular labile heme pool (LHP) [12]. Consequently, it is unlikely that free heme exists at concentrations high enough to facilitate passive diffusion through the pore structures of the parasitophorous vacuole membrane (PVM), like TgGRA17 and TgGRA23 [13]. While *Toxoplasma* is known to ingest host cytoplasmic proteins and deliver them to the plant-like vacuolar compartment (PLVAC), a lysosome-like digestive organelle [14], it is a plausible hypothesis that the parasites also internalize host hemoproteins and liberate heme within the PLVAC (Fig 1A), though a dedicated heme transporter in this organelle has yet to be identified.

The observation that heme-deficient strains (∆*cpox,* ∆*ppo*, and ∆*alas*) can propagate in standard medium, albeit with reduced growth rates, suggests that these parasites utilize host-derived heme precursors. This hypothesis is reinforced by the fact that exogenous ALA partially rescues growth defects in these strains. Mechanistically, while the CPOX-catalyzed decarboxylation of coproporphyrinogen III has a high activation energy and does not proceed spontaneously, the subsequent conversion of protoporphyrinogen IX (PPgen IX) to protoporphyrin IX (PPIX) can occur via auto-oxidation. Furthermore, because host cells accumulate significant levels of PPIX in the presence of ALA [8], it is speculated that *Toxoplasma* scavenges host-derived PPgen IX or PPIX for growth rescue (Fig 1A). The dramatic increase of PPIX in ∆*cpox* parasites supports this idea [8], suggesting a strategy of acquiring lipophilic intermediates via passive diffusion. In contrast, the failure of exogenous heme to rescue ∆*cpox* or TgFECH knockdown strains suggests that *Toxoplasma* lacks a specific heme transporter and cannot directly access the exogenous heme pool [6,8].

## Is the pathway druggable?

Early pharmacological studies showed that heme biosynthesis inhibitors, including succinylacetone and the diphenyl ether herbicides acifluorfen and oxyfluorfen, impair parasite growth *in vitro* [15]. However, since these compounds also affect host heme production, they could not distinguish between inhibition of the parasite pathway and disruption of host-derived heme supply. The recent genetic disruption of heme biosynthesis results in a drastic loss of acute virulence in a murine model [6], establishing the pathway as a potential target for therapeutic intervention. The presence of plant-like enzymes in the parasite pathway makes it particularly attractive for drug development. Phylogenetic placement of TgPPO within the plant clade, distinct from the mammalian ortholog (Fig 1B) [6], reinforces this rationale for selective inhibitor development. PPO is a well-validated target of commercial herbicides known for their high potency against plants and low mammalian toxicity [16], and the *Toxoplasma* ortholog is phylogenetically closer to plant orthologs than to mammalian counterparts [6]. Five commercially available PPO-targeting herbicides were shown to inhibit parasite growth at sub-millimolar concentrations [6]. Chemical modification of the most potent compound, oxadiazon, lowered the half-maximal effective concentration (EC_50_) to below 10 µM [17,18]. Although these potencies remain modest compared to frontline therapeutics such as pyrimethamine, they represent a promising starting point for medicinal chemistry optimization. Structure-guided drug design, informed by three-dimensional structures of parasite-specific enzymes, could substantially improve both selectivity and potency. Additional pathway enzymes, particularly those localized to the apicoplast, may also warrant exploration as drug targets.

These insights extend to other apicomplexan parasites that use diverse strategies for heme acquisition [10]. *Plasmodium* dispenses with *de novo* synthesis during asexual blood stages, scavenging heme from host hemoglobin instead, but depends on the *de novo* pathway during mosquito and liver stages. *Cryptosporidium* has lost the pathway entirely, whereas *Babesia* and *Theileria* retain *Toxoplasma*-like architectures [10]. Selective inhibitors developed against *Toxoplasma* could therefore inform drug discovery against pre-erythrocytic malaria and the economically important piroplasmid infections of livestock.

## What is the relationship between heme metabolism and artemisinin resistance?

Artemisinin and its derivative dihydroartemisinin (DHA) are widely thought to require activation by heme or ferrous iron, making intracellular heme availability a key determinant of drug efficacy [19,20]. Two recent studies have linked heme biosynthesis to artemisinin sensitivity in *Toxoplasma* [20,21]. A genome-wide CRISPR knockout screen identified genes in the TCA cycle and heme biosynthesis pathway as critical modulators of DHA susceptibility [20]. Consistent with this, chemical inhibition of heme biosynthesis by succinylacetone reduced intracellular heme and porphyrin levels and significantly increased the DHA EC_50_ [20]. Genetic disruption of DegP2, a mitochondrial protease identified in the screen, similarly lowered free heme and conferred DHA resistance [20]. An independent study using *in vitro* resistance evolution also identified a point mutation in DegP2 among parasites with moderately increased DHA tolerance, alongside mutations in a kinase of unknown function (Ark1) and amplification of mitochondrial DNA encoding cytochrome oxidase subunits [21]. However, targeted deletion of DegP2 and Ark1, alone or in combination, conferred only modest resistance to DHA [21], suggesting that multiple nuclear- and mitochondrial-encoded factors contribute to the resistance phenotype.

These findings have an important implication for therapeutic design. Because DHA activation depends on intracellular heme, pharmacological inhibition of heme biosynthesis would be predicted to antagonize, rather than synergize with, artemisinin derivatives. Direct measurement of DHA sensitivity in heme-deficient mutants (*e.g.,* Δ*cpox* and Δ*ppo*) would provide a quantitative assessment linking heme availability to artemisinin resistance. If confirmed, heme pathway inhibitors should be paired with mechanistically distinct partner drugs rather than with artemisinins, and co-administration should be approached cautiously in any future clinical setting.

## Future directions

The role of heme metabolism during chronic *Toxoplasma* infection remains largely unexplored. Transcriptomic data indicate that all eight heme biosynthetic genes are downregulated during the tachyzoite-to-bradyzoite transition [22], suggesting reduced reliance on *de novo* heme synthesis during chronic infection. Heme-deficient mutants display markedly reduced acute virulence, raising the question of whether they can disseminate to the central nervous system and initiate cyst formation. Therefore, conditional genetic ablation of heme biosynthetic genes in cyst-forming strains (*e.g.*, ME49 or Pru) will be needed to resolve this question. Importantly, transcriptional downregulation of heme biosynthetic genes is not direct evidence that *de novo* heme production is dispensable during chronic toxoplasmosis. Bradyzoites retain mitochondrial activity and detectable heme [23], implying that a basal heme flux is sustained even in chronic stages. Moreover, the bradyzoite-to-tachyzoite reactivation that drives clinical recrudescence is accompanied by re-engagement of oxidative metabolism, which would require heme biosynthesis to resume. Targeting this pathway may therefore be most relevant for acute infection and for blocking reactivation, rather than for eliminating established cysts. Whether residual heme biosynthetic flux in bradyzoites is sufficient for pharmacological modulation remains an open question that conditional ablation studies will help resolve. Further medicinal chemistry optimization of existing PPO inhibitors, guided by three-dimensional enzyme structures, could yield compounds with improved potency and selectivity. Collectively, these findings position heme biosynthesis as a promising and underexploited anti-*Toxoplasma* drug target.
